# Health in Our Hands: Harnessing the Power of Lifestyle Medicine

**DOI:** 10.3389/ijph.2025.1607670

**Published:** 2025-02-11

**Authors:** Manuel Weber

**Affiliations:** ^1^ Epidemiology, Biostatistics and Prevention Institute, University of Zurich, Zurich, Switzerland; ^2^ Academic-Practice-Partnership Between School of Health Professions at Bern University of Applied Sciences and University Hospital of Bern, Bern University of Applied Sciences, Bern, Switzerland; ^3^ Swiss School of Public Health, Zurich, Switzerland

**Keywords:** health promotion, health-related quality of life, life expectancy, non-communicable disease, prevention

**The IJPH series “Young Researcher Editorial” is a training project of the Swiss School of Public Health**.

Are our daily lifestyle choices the key to longer and healthier lives? Although life expectancy continues to rise due to advances in healthcare and better living conditions, these added years may not be lived in good health. Recently, global healthy life expectancy (HALE) at birth has lagged behind overall life expectancy at birth [1]. While the latter is expected to increase by 4.6 years from 2022 to 2050, global HALE at birth is only anticipated to grow by 3.1 years [2].

A key driver of the growing disparity between life expectancy and HALE is the rising burden of non-communicable diseases (NCDs) such as cardiovascular and respiratory diseases, diabetes, cancer, and mental disorders [1]. Between 2010 and 2021, global disability-adjusted life years from NCDs increased by 17.6% [3] and the global burden of disease will likely continue to shift towards NCDs [2]. NCDs raise both direct and indirect healthcare costs [4], so health systems worldwide must pivot to reduce the disparity between life expectancy and HALE and halt the rise in NCDs. Health systems should take a comprehensive approach to devising strategies for disease prevention, early detection, and effective disease management.

Health systems may be able to relieve some of this burden by promoting lifestyle medicine (LM). LM is an evidence-based approach to preventing and managing diseases, resting on six pillars: nutrition; physical activity; restorative sleep; avoidance of risky substances; social connection; and stress management [5]. These are modifiable lifestyle behaviors that, along with others such as sexual behavior, influence our health and well-being. As a healthy lifestyle rests on all these pillars, people benefit from increasing multiple healthy lifestyle behaviors. A greater number of poor lifestyle behaviors was associated with a higher prevalence of poor health-related quality of life [6]. Conversely, engaging in a greater number of positive lifestyle behaviors was associated with more disease-free years in individuals free of major NCDs, regardless of socioeconomic status [7]. In chronic disease management, a recent systematic review and meta-analysis of 43 randomized controlled trials found small to large positive effects (*d* = 0.081–2.003) of interventions targeting multiple lifestyle behaviors (except for smoking: *d* = −0.019) [8].

LM could be extensively applied in health promotion, disease prevention, and disease management. By holistically addressing multiple lifestyle pillars and centering on an individual’s needs and resources, LM offers a proactive and interdisciplinary approach to health and well-being. Behavioral changes in lifestyle pillars can be addressed sequentially or simultaneously, depending upon an individual’s needs and circumstances.

Lifestyle choices can have profound effects on health and well-being. In mental health, for example, a systematic meta-review of meta-analyses, Mendelian randomization studies, and meta-reviews underscored the critical role of physical activity, diet, smoking, and sleep in preventing and treating mental disorders (e.g., depression) [9]. Furthermore, adherence to a healthy lifestyle at mid-life was associated with increased overall life expectancy and a greater number of years free of major chronic diseases in a prospective cohort study; specifically, healthy lifestyle was associated with a lower risk of cardiovascular diseases, type 2 diabetes, and cancer [10]. Additionally, LM approaches may also reduce healthcare costs and improve employee productivity [11].

Healthy lifestyle alone cannot prevent diseases with genetic components, since both genetic and lifestyle behaviors influence lifespan independently. But a recent longitudinal cohort study using data from three large population-based cohorts revealed that adhering to healthy lifestyles could mitigate genetic risk of a shorter lifespan or premature death [12]. Adopting the optimal combination of lifestyle behaviors, including a healthy diet, regular physical activity, adequate sleep duration, and never smoking, could therefore provide substantial longevity benefits, regardless of genetic background.

Much of our health and well-being is in our hands, so we must take responsibility for proactively managing them. We must learn how lifestyle choices affect our health and adopt healthy lifestyle habits, so society – through healthcare systems and supportive policies – should educate and instruct us in methods for improving our health and well-being through informed lifestyle choices and better self-management [13]. But populations cannot do that unless governments implement effective public health strategies and interventions that harness the power of LM.

Governments should increase their investment in preventive measures and health promotion activities and fund interdisciplinary initiatives that involve health professionals from various lifestyle-related disciplines. Such a comprehensive approach aligns with the principles of LM. Public health should leverage the potential of LM to bridge the widening gap between life expectancy and HALE and to address the rising burden of NCDs. Integrating LM into health promotion, disease prevention, and management strategies could promote healthy longevity and improve health-related quality of life.

## Abbreviations

HALE, healthy life expectancy; LM, lifestyle medicine; NCD, non-communicable disease.
